# A Role for Cytosolic Isocitrate Dehydrogenase as a Negative Regulator of Glucose Signaling for Insulin Secretion in Pancreatic ß-Cells

**DOI:** 10.1371/journal.pone.0077097

**Published:** 2013-10-10

**Authors:** Claudiane Guay, Érik Joly, Émilie Pepin, Annie Barbeau, Lisa Hentsch, Marco Pineda, S. R. Murthy Madiraju, Henri Brunengraber, Marc Prentki

**Affiliations:** 1 Molecular Nutrition Unit and the Montreal Diabetes Research Center at the Centre de Recherche du Centre Hospitalier de l'Université de Montréal, CR-CHUM, Montreal, Quebec, Canada; 2 Departments of Nutrition and Biochemistry, University of Montreal, Montreal, Quebec, Canada; 3 Department of Nutrition, School of Medicine, Case Western Reserve University, Cleveland, Ohio, United State of America; Boston University, United States of America

## Abstract

Cytosolic NADPH may act as one of the signals that couple glucose metabolism to insulin secretion in the pancreatic ß-cell. NADPH levels in the cytoplasm are largely controlled by the cytosolic isoforms of malic enzyme and isocitrate dehydrogenase (IDHc). Some studies have provided evidence for a role of malic enzyme in glucose-induced insulin secretion (GIIS) via pyruvate cycling, but the role of IDHc in ß-cell signaling is unsettled. IDHc is an established component of the isocitrate/α–ketoglutarate shuttle that transfers reducing equivalents (NADPH) from the mitochondrion to the cytosol. This shuttle is energy consuming since it is coupled to nicotinamide nucleotide transhydrogenase that uses the mitochondrial proton gradient to produce mitochondrial NADPH and NAD^+^ from NADP^+^ and NADH. To determine whether flux through IDHc is positively or negatively linked to GIIS, we performed RNAi knockdown experiments in ß-cells. Reduced IDHc expression in INS 832/13 cells and isolated rat islet ß-cells resulted in enhanced GIIS. This effect was mediated at least in part via the K_ATP_-independent amplification arm of GIIS. IDHc knockdown in INS 832/13 cells did not alter glucose oxidation but it reduced fatty acid oxidation and increased lipogenesis from glucose. Metabolome profiling in INS 832/13 cells showed that IDHc knockdown increased isocitrate and NADP^+^ levels. It also increased the cellular contents of several metabolites linked to GIIS, in particular some Krebs cycle intermediates, acetyl-CoA, glutamate, cAMP and ATP. The results identify IDHc as a component of the emerging pathways that negatively regulate GIIS.

## Introduction

The secretion of insulin by pancreatic β-cells in response to glucose and other stimuli regulates fuel homeostasis [1]. However, the pathways involved in glucose-induced insulin secretion (GIIS) remain to be ascertained. A triggering pathway links insulin vesicle exocytosis to glucose metabolism via an elevation in cytosolic ATP, closure of the K_ATP_ channels, depolarisation of the plasma membrane and the opening of voltage-gated Ca^2+^ channels [2], [3], [4], [5]. However, K_ATP_/Ca^2+^-independent amplification pathways are also involved in GIIS [6]. Metabolic coupling factors derived from glucose metabolism, in particular ATP [3], malonyl-CoA [7], [8], other short-chain acyl-CoAs [9], glutamate [10], mitochondrial GTP [11], reactive oxygen species [12], [13] and NADPH [14], [15], have been proposed to be critical players in GIIS [16].

Mitochondrial metabolism is an important component of GIIS [17]. Nearly 50% of the pyruvate derived from glycolysis in rodent β-cells enters the Krebs cycle via conversion to oxaloacetate via pyruvate carboxylase (PC) [18], [19]. This anaplerotic process leads to the net synthesis and accumulation of Krebs cycle intermediates and is coupled to the export (cataplerosis) of metabolites such as malate and citrate [20], [21] into the cytosol where their metabolism leads to the production of signaling molecules for insulin secretion. We and others have studied the role of anaplerosis in GIIS [20], [21], [22], [23] and documented the significance of three pyruvate cycles in the regulation of GIIS: the pyruvate/malate [14], [24], [25], pyruvate/citrate [21], [26] and the so-called pyruvate/isocitrate/α-ketoglutarate cycle [27].

Only pyruvate/citrate cycling leads to malonyl-CoA formation for lipid signaling and the production of cytosolic NAD^+^ needed for fast glycolytic flux [21], [26]. However, all these three cycles produce NADPH in the cytosol [14], [26], [27]. In the pyruvate/malate and pyruvate/citrate cycles, NADPH is produced by cytosolic malic enzyme (MEc). In the pyruvate/isocitrate/α-ketoglutarate cycle cytosolic isocitrate dehydrogenase isozyme-1 (IDHc; IDH1) is responsible for NADPH production. Since the pentose-phosphate pathway is not quantitatively significant in normal islet β-cells [20], [28], MEc and IDHc activities are the major source of cytosolic NADPH. Interestingly, the dose dependence of GIIS correlates with the cellular NADPH/NADP^+^ ratio [15], [25], [27], [29]. Also, NADPH directly stimulates exocytosis of insulin granules in patch clamped ß-cells [15]. Thus, NADPH produced in the β-cell cytosol by MEc and/or IDHc may act as a metabolic coupling factor for insulin secretion, possibly via glutaredoxin-1 [30].

Several studies, but not all [31], have shown that reduction of MEc expression in INS 832/13 cells, MIN6 cells and in mouse islets impaired GIIS and correlated with a decrease in NADPH level [24], [25], [26], [32]. In rat islets, siRNA knockdown of MEc mRNA did not affect GIIS. However, the level of islet MEc protein and the NADPH/NADP^+^ ratio were not measured in this study [32]. Knockdown of IDHc expression using a siRNA approach with adenoviral constructs has been shown in one study only to reduce GIIS in INS 832/13 cells and rat islets. There was also a modest 15% decrease in NADPH/NADP^+^ ratio at elevated glucose and a decrease in NADP^+^ rather than an elevation as anticipated [27]. This study favored the view that IDHc plays critical role in GIIS [27]. The results of this study, however, have not been confirmed by other groups. In view of our interest in pyruvate cycling processes, we tested if down regulating simultaneously malic enzyme and IDHc could have additive inhibitory effect on GIIS. However, we could not reproduce the observation that IDHc knockdown inhibits GIIS. Therefore we decided to reexamine the role of this enzyme in the coupling mechanisms of GIIS.

In the proposed “pyruvate/isocitrate/α-ketoglutarate cycle” [27], IDHc converts cytoplasmic isocitrate to α-ketoglutarate and NADP^+^ to NADPH. α-Ketoglutarate enters mitochondria and is converted back to pyruvate via steps of the pyruvate/malate cycle or via mitochondrial malic enzyme. Thus, this cycle, unlike the two others, is not an independent one and may not be appropriately termed an anaplerotic pyruvate cycling process. In other tissues, it is a well-known loop for the reversible transfer of NADPH between the cytosolic and mitochondrial compartments [33], [34], [35]. We prefer to name it for what it has traditionally been known: an isocitrate/α-ketoglutarate NADPH shuttle. Interestingly, this shuttle is energy consuming since it has been established that it is coupled to nicotinamide nucleotide transhydrogenase that uses the mitochondrial proton gradient to produce mitochondrial NADPH and NAD^+^ from NADP^+^ and NADH [34]. Thus, nicotinamide nucleotide transhydrogenase is coupled to NADPH-dependent isocitrate dehydrogenase isozyme-2 (IDH2) and IDH2 in turn is coupled to cytosolic IDH (IDHc) (see also figure 6). Importantly, these points had not been realized in the ß-cell glucose signaling literature dealing with IDHc or pyruvate cycling processes, but we have reviewed them recently [1].

In the present study, we reinvestigated the role of IDHc in the regulation of GIIS using a non-viral-based siRNA delivery approach (transfected cells). We found that decreased IDHc expression in INS 832/13 cells and isolated rat islet ß-cells resulted in enhanced GIIS. Knockdown of IDHc in INS 832/13 cells was associated with enhanced anaplerosis and acetyl-CoA levels and increased production of established and candidate metabolic coupling factors for insulin secretion. Overall the data indicate that IDHc is not involved in stimulatory pathways for GIIS but is rather a component of the emerging negative/decelerating modulatory pathways of fuel signaling.

## Materials and Methods

***Cell Culture*** - Rat insulinoma INS 832/13 cells [36] (passages 51–64) were grown in monolayer and cultured in RPMI 1640 medium at 11.1 mM glucose supplemented with 10% (v/v) fetal bovine serum, 10 mM HEPES, 2 mM L-glutamine, 1 mM sodium pyruvate and 50 µM β-mercaptoethanol (referred as complete RPMI) at 37°C in a humidified atmosphere (5% CO_2_, 95% air).

***Islet Isolation*** - All animal procedures were performed in accordance with NIH guidelines and the protocol was approved by the institutional committee for the protection of animals at the Centre de Recherche du Centre Hospitalier de l'Université de Montréal (Permit number An09059MPr). Wistar rats (200–250 g) were obtained from Charles River (St. Constant, QC, Canada). Rats were anesthetized with sodium pentobarbital (Somnotol, MTC Pharmaceuticals, Hamilton, ON, Canada) and killed by exsanguination. All efforts were made to minimize suffering. Pancreatic islets were isolated by collagenase digestion of the pancreas [37]. After digestion and washing, islets were separated from digested exocrine tissue by histopaque gradient, after which they were hand-picked. Isolated islets were incubated overnight in complete RPMI at 11 mM glucose containing 100 µg/mL of streptomycin and 100 IU/mL of penicillin without β-mercaptoethanol at 37°C in a humidified atmosphere (5% CO_2_, 95% air).

***siRNA Mediated Gene Suppression*** – siRNAs directed against rat cytosolic NADP^+^-dependant isocitrate dehydrogenase (IDH1 isoform, GenBank™ accession number NM_031510) were purchased from Ambion® (Applied Biosystems, Streetsville, ON, Canada). siRNA duplexes were targeted to 21-pb regions of the IDHc cDNA sequence beginning at nucleotides 73, 97 and 282 and are referred as siIDH#1, siIDH#2 and siIDH#3, respectively. The combination of two different siRNA with no known target provided by Ambion as negative control was used as the siRNA Scrambled. The Silencer Negative Control#A and #B sequences were 5′-UAA CGA CGC GAC GAC GUA Att-3′ and 5′-UCG UAA GUA AGC GCA ACC Ctt-3′, respectively and referred as ScrAB. The purity of each siRNA strand was measured by analytical HPLC and guaranteed by Ambion.

***Cell Transfection*** – Cells were seeded in 75-cm^2^ flasks at 4×10^6^ cells two days prior to transfection and were at 60–70% confluence at the time of transfection. siRNA duplexes were introduced into INS 832/13 cells using the Nucleofector technology (Amaxa Inc., Walkersville, MD, USA) [26]. To preserve a ratio of 5 µg of DNA for 6×10^6^ cells, 4.4 µg of plasmid pBlueScriptII (Agilent Technologies, ON Canada) were combined to 0.6 µg of siRNA. A control condition with plasmid BlueScript alone transfected cells (referred as pBS) was included. After transfection, INS 832/13 cells were seeded in: 12-well plates at 6×10^5^ cells for insulin secretion assays, protein content, and mRNA analysis; 6-well plates at 1.6×10^6^ cells for glucose incorporation into total lipids assay; 60-mm tissue culture plates at 2.5×10^6^ cells for IDHc activity determination; in 25-cm^2^ flasks at 3.2×10^6^ cells for glucose and fatty acid oxidation measurements; and in 100-mm tissue culture at 5×10^6^ cells for malonyl-CoA and metabolite determinations. Experiments were performed 48 h post-transfection and the final density was equivalent between all the experiments performed.

***Cell Infection*** – Cells were treated as described below in order to reproduce as closely as possible the method used by C. Newgard and coll. for INS 832/13 cells [27], [32], [38], [39], [40]. Cells were seeded in 12-well plates at 2×10^5^ cells/well and cultured for 36 h before infection. Cells were infected with adenoviruses expressing the bacterial β-galactosidase under the beta-actin promoter (Ad-LacZ) [41] or expressing GFP under the CMV-5 promoter (Ad-GFP) (kindly provided by Dr Yves Langelier [42]) at multiplicity of infection (MOI) of viral dose of 10 plaque-forming units (pfu)/cell for 16 h in 0.5 mL of complete RPMI. They were then washed and further cultured for 48 h in 1 mL of the same media prior to insulin secretion experiments. The adenoviruses were amplified using the packaging cell line BMAd200-8, and purified by CsCl ultracentrifugation. CsCl was removed by dialysis in 20 mM Tris pH 8.0, 25 mM NaCl and 2.5% glycerol, and viral aliquots were stored at −80°C for single use. Stock titer was carefully assessed using the plaque-assay method with the permissive 293A cell line (ATCC, Manassas, VA).

***Real-Time Quantitative PCR Analysis*** –RNA cell content was extracted using the RNeasy Mini Kit (Qiagen, Mississauga, ON, Canada) with RNase-Free DNase (Qiagen). RNA was reversed transcribed to cDNA using MMLV reverse transcriptase (Invitrogen, Burlington, ON, Canada) and hexamers as described previously [43]. Forward and reverse sequence primers (IDT, Coralville, IA) were designed specifically for IDHc and mRNA levels were normalized to cyclophilin and to 18S. Primer sequences were: IDHc F: 5′-CTG GAT CTG CAT AGC TAT GAC-3′, and R: 5′-CCT CAA CCC TCT TCT CAT CG-3′. Cyclophilin F: 5′-CTT GCT GCA GAC ATG GTC AAC-3′, and R: 5′-GCC ATT ATG GCG TGT GAA GTC-3′.18S F: 5′-CTG AGA AAC GGC TAC CAC ATC-3′, and R: 5′-GGC CTC GAA AGA GTC CTG TAT-3′. Real-time quantitative PCR were performed on Rotor-Gene RG-3000 (Corbett Research, Mortlake, NSW, Australia) using LC Faststart DNA Master^plus^ SYBR Green reagent (Roche, Laval, QC, Canada). Results were analyzed using the Rotor-Gene software version 6.0.19 provided by Corbett Research.

***Cytosolic NADP^+^-Dependant Isocitrate Dehydrogenase 1 Activity*** – Isocitrate dehydrogenase activity was assayed in fresh cytosolic cell extracts. Briefly, 48 h post-transfection, cells were placed on ice, washed with cold PBS, extracted by scraping in ice-cold PBS/EGTA buffer containing protease inhibitors (1 µg/ml of leupeptin and pepstatin, 1 mM phenylmethylsulfonylfluoride, and 1.5 µg/ml of aprotinin; referred as protease inhibitor mix). Cell suspension was centrifuged at 1,000× *g* for 2 min at 4°C. The cell pellet was resuspended in 500 µl of an ice-cold homogenization buffer containing 20 mM HEPES (pH 7.4), 0.25 M sucrose, 1 mM EGTA, and protease inhibitor mix, and homogenized by hand using a 1 ml Potter-Elvehjem homogenizer with 25 gentle passes. Homogenates were centrifuged at 1,000× *g* for 2 min at 4°C to discard nuclei and then at 12,000× *g* for 30 min at 4°C to discard mitochondria. The post-mitochondrial supernatant content was concentrated using a Microcon® YM-30 (Millipore, Billerica, MA). The final volume of the concentrate was adjusted to 100 µl with ice-cold homogenization buffer (see above), and 40 µl was used for IDH activity. The assay system in 96-well plates contained 100 mM of Tris buffer (pH 8.0), 7% glycerol, 3 mM MgCl_2_, 1 mM NADP^+^ and 3 mM of isocitrate. Reactions were started by adding cytosolic extracts, immediately followed by shaking, and absorbance at 340 nm was read every 15 s for up to 10 min using Fluostar Optima (BMG Labtechnologies, Offenburg, Germany). A blank control was run without isocitrate. Enzymatic activity was determined by measuring the rate of NADPH production. Activities were normalized to protein content using a BCA protein kit (Pierce, Rockford, IL).

***Insulin Secretion and Content***– siRNA-transfected INS832/13 cells were pre-incubated during 2 h in RPMI complete medium at 1 mM glucose and then were equilibrated for 1 h in Krebs-Ringer bicarbonate buffer containing 10 mM HEPES (KRBH; pH 7.4), 0.5% defatted BSA (Sigma), and 1 mM glucose. Cells were then incubated for 45 min in KRBH containing 0.5% BSA and 1, 5 or 10 mM glucose, in the presence or absence of 35 mM KCl, and/or 20 µM diazoxide. Stock solution of diazoxide was prepared in DMSO and final DMSO concentration in KRBH was 0.5%. At the end of the incubations, media were collected for insulin determination. Total insulin content of the cells was measured after acid-ethanol (0.2 mM HCl in 75% ethanol) extraction. Insulin levels were measured by radioimmunoassay using a human insulin standard (Linco Research, St. Charles, MO). Insulin secretion was normalized to total cellular insulin content or protein content.

***Glucose and Fatty Acid Metabolism*** – Glucose oxidation to CO_2_, fatty acid oxidation to CO_2_ and acid-soluble products [44] and glucose incorporation into fatty acids were measured in transfected INS 832/13 cells pre-incubated as described for insulin secretion experiments. For glucose oxidation determinations, cells were incubated for 45 min in KRBH containing 0.5% BSA and, 1 mM glucose with 0.1 µCi/ml of [U-^14^C]glucose (GE Healthcare, Baie d'Urfé, QC, Canada), or 10 mM glucose and 0.2 µCi/ml of [U-^14^C]glucose. The experiments were performed as previously described [26]. For fatty acid oxidation experiments, cells were incubated for 45 min in KRBH containing 0.5% BSA, 1, 5 or 10 mM glucose in the presence of 1 mM carnitine plus 0.2 mM palmitate, and 0.1 µCi/ml of [1-^14^C]palmitate (PerkinElmer Life Sciences, Downers Grove, IL). Beta-oxidation to CO_2_ and acid soluble products was performed as previously described [45]. For the measurement of glucose incorporation into the fatty acid moiety, cells were incubated for 2 h in KRBH containing 0.5% BSA, at 1, 5 or 10 mM glucose and with 3 µCi/ml [U-^14^C]glucose in the presence of 1 mM oleate. At the end of the incubations, media were removed, cells were washed once with PBS and 1 ml of PBS containing 0.53 mM EDTA was added to the plated cells. Cells were collected with gentle pipetting, centrifuged at 500× *g* for 10 min at 4°C, dissolved in 3 ml of CHCl_3_∶methanol∶HCl (200∶100∶1 v/v), and placed at −20°C until the extraction. Lipid extractions and analysis of ^14^C-fatty acids were performed as previously described [26].

***Malonyl-CoA Measurements*** – Malonyl-CoA levels were measured in transfected INS 832/13 cells pre-incubated and incubated as described for insulin secretion assay. After the incubation, media were discarded and malonyl-CoA was extracted using 10% trichloroacetic acid [46]. Cell extracts were centrifuged at 10,000× *g* for 5 min at 4°C to precipitate proteins. The supernatant was collected and washed with successive ether extractions to neutralize the extract pH. Samples were lyophilized and stored at −80°C. Malonyl-CoA levels were determined based on a radioactive method using [U-^3^H]acetyl-CoA and fatty acid synthase [47].

***Rat Islets Dispersion and Transfection*** – After overnight incubation in complete RPMI (see islet isolation), islet cells were dispersed by incubating islets in Ca^2+^/Mg^2+^ free phosphate buffered saline, 3 mM EGTA and 0.002% trypsin for 5 min at 37°C with gentle shaking by pipetting. Dispersed cells (1.4×10^6^ cells per condition) were transfected using Nucleofactor technology (Amaxa) with 800 nM of siRNA (ScrAB or siIDH#2) and 5 µg of the human growth hormone (hGH) (Open Biosystems, Thermo Fisher Scientific, Huntsville, AL) used as a reporter of insulin secretion in transfected cells [48], [49]. After transfection, dispersed islet cells were seeded in 24-well plates at 1.2×10^5^ cells. Experiments were performed 48 h post-transfection.

***Insulin Secretion on Transfected Dispersed Islet Cells*** – siRNA-transfected dispersed rat islet cells were pre-incubated for 2 h in RPMI complete medium without β-mercaptoethanol at 2 mM glucose and then equilibrated for 1 h in KRBH containing 0.5% BSA and 2 mM glucose. Cells were then incubated for 1 h in KRBH containing 0.5% BSA and 2, 8 or 16 mM glucose, and at 2 mmol/L glucose with 35 mM KCl. At the end of the incubation period, media were collected for hGH determination. Total hGH of the cells were measured after acid-ethanol extraction as described above. Cells were also harvested with urea lysis buffer (6 M urea, 2% SDS, 62.5 mM Tris-HCl) for total protein content determination. Total hGH content and hGH release were measured by a colorimetric enzyme immunoassay (Roche). hGH levels was normalized to total hGH content, and to protein content.

***Metabolite Extraction and Targeted Metabolomic***– Six-well culture plates were seeded with siRNA-transfected INS 832/13 cells at a density of 3×10^5^ cells per well. Metabolite extraction was performed 48 h after transfection as follow. First, cells were pre-incubated in RPMI containing 2 mM glucose, followed by 2 successive incubations in KRBH media, exactly as described under the section of insulin secretion for INS 832/13 cells. At the end of the incubation period with low or high glucose concentration, media were rapidly aspirated and culture plates with attached cells were frozen in a liquid nitrogen bath for 2 min. The plates were then transferred on ice and 530 µL of ice-cold extraction buffer (80% methanol, 10 mM ammonium acetate pH 9) were added to the wells. Each well was also spiked with stable isotope internal standards (AMP, 4 µM adenosine-^13^C10,^15^N5 5′-monophosphate; citrate, 5 µM citric acid-^13^C6 and non-hydrolysable ATP, 10 µM adenosine 5′-(β,γ-imido) triphosphate) (Sigma Aldrich, St. Louis, MO). Cells were then scraped off the plates and transferred into microcentrifuge tubes kept on ice. A volume of 150 µL of extraction buffer was used to rinse each well and combined to homogenate. All homogenates were sonicated in a cup-horn sonicator at maximal power for 2 min in a water-ice bath and centrifuged at 4°C for 10 min at 15,000× g. Supernatants were collected into ice-cold glass tubes, while pellets were washed with 150 µL of fresh extraction buffer solution, vortexed and centrifuged as described above. Corresponding supernatants were combined into glass tubes and two volumes of chloroform were added. Tubes were then mixed vigorously and centrifuged at 13,000× g for 20 min at 4°C. The upper aqueous phases were collected carefully without carrying out any interface material and transferred into cold glass tubes and subjected to another chloroform extraction. After centrifugation as above, 200 µL of the upper aqueous phases were transferred into polypropylene tubes, frozen in liquid nitrogen, lyophilized until dryness (4–6 h) in a FreezeMobile 25EL lyophilizer (Virtis Company, NY) and stored at −80°C until LC-MS/MS analysis.

To analyze the samples on LC/MS/MS, we devised a new method, based on three different publications [50], [51], [52]. This allowed measurements of different classes of metabolites (forty altogether) in a single 13 min run on a liquid chromatography electrospray ionization tandem mass spectrometry system (LC-ESI-MS/MS). Samples were reconstituted in 12 µL of water, and injections of 3 µL were performed in triplicate on a LC–MS/MS system composed of an Agilent 1200 SL (LC) and a triple quadrupole mass spectrometer (4000Q TRAP MS/MS, AB Sciex). Samples were separated on a Zorbax Eclipse Plus-C18 (2.1×50 mm, 3.5 µm) column (Agilent Technologies). The mobile phase consisted of solvent A (10 mM of the ion pairing agent tributylamine, 15 mM acetic acid, pH 4.95) and solvent B (methanol) at a flow rate of 0.5 mL/min with a column temperature of 40°C. The MS was operated in negative electrospray ionization mode using a turbo ion spray source. Quantification was performed by integrating peak areas from the selected transition using Multiquan software (version 2.0.1; AB Sciex), and subsequently all metabolite peaks were manually reviewed for peak quality prior to analysis. Metabolite levels were determined using a specific standard curve for each metabolite. To precisely determine the percentage of recovery of the individual metabolite, a known concentration of each metabolite standard (between 0.05 and 2 nmol, depending on the metabolite) was spiked in the incubation media, prior to the extraction (or in KRBH for blank controls). Metabolite recoveries were found to be in the range of 60 to 98%, except for glutamine (10–15%). Importantly, metabolite recoveries were highly reproducible, even for those with lower yield, including glutamine. Recovery rates were taken into account for the determination of the absolute amount of each metabolite. Finally, protein contents were determined from cells transfected and plated in parallel wells and measured as described above using the BCA assay.

***Statistical Analysis*** – Data are expressed as means ± SEM. Statistical significance was calculated with the Student's *t* test or, for multiple comparisons, one-way analysis of variance (ANOVA) with Dunnett's post-test as indicated. A *p* value of <0.05 was considered significant. Statistical analyses were performed using InStat program (GraphPad Software, San Diego, CA).

## Results

***Impact of the siRNA Delivery Methods on Insulin Secretion*** – The RNAi technology has been used to investigate the role of anaplerosis and pyruvate cycling processes in GIIS [24], [25], [26], [27], [32], [38], [39], [40], [53], [54]. Normal ß-cells and INS 832/13 ß-cells are difficult to transfect and therefore earlier studies generally used adenoviral vectors to deliver RNAi or cDNA into normal rodent islet cells as well as in INS1 cells and INS 832/13 cells. Under our experimental conditions, we observed that the transfection efficiency of INS 832/13 cells using the Nucleofactor technology (Amaxa©) is approximately 85% based on GFP expression. In order to choose the optimal technology to study ß-cell metabolic fuel signaling we compared two RNAi delivery methods in term of basal and GIIS: Nucleofactor transfection and adenoviral infection.

**Figure 1:**
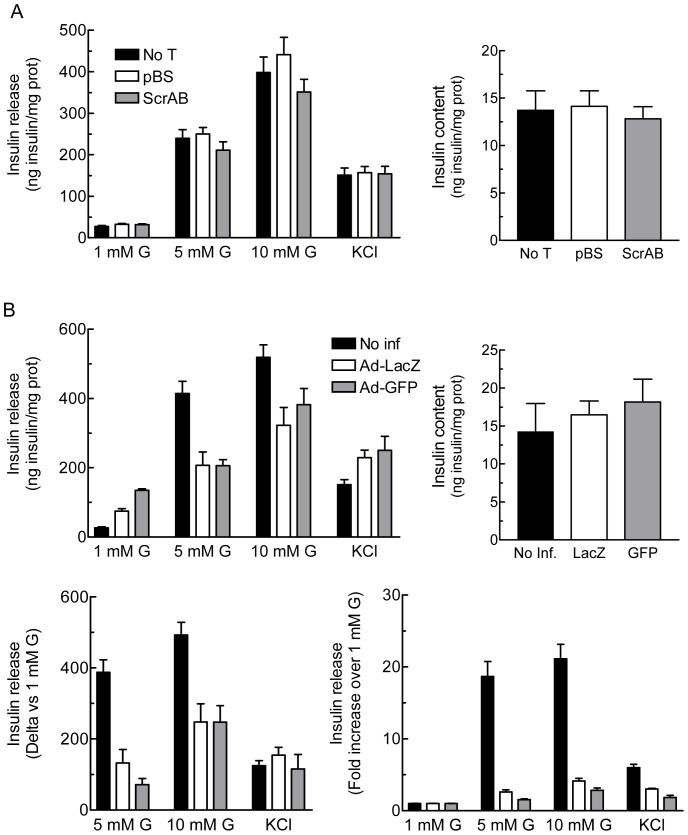
Effect of the RNA interference delivery method on glucose-induced insulin secretion in INS 832/13 cells. A, Glucose-induced insulin secretion is not affected by Nucleofactor transfection. INS 832/13 cells were not transfected (No T) or transfected with an empty vector (pBS) or a combination of two siRNA controls (ScrAB) using Nucleofactor electroporation. Cells were cultured for 48 h prior to the experiment. B, Adenoviral infection *per se* alters glucose-induced insulin secretion. INS 832/13 cells were not infected (No inf) or infected with one control adenovirus containing LacZ or GFP (Ad-LacZ or Ad-GFP) at 10 MOI for 16 h. After the infection period, cells were cultured for 48 h prior to the experiment. Insulin release was measured in cells incubated at 1, 5 or 10 mM glucose (G) or 1 mM glucose plus 35 mM KCl. Insulin levels were normalized by protein content. Data represent the mean ± SEM of two to three independent experiments performed in quadruplicate.

INS 832/13 cells transfected with a control siRNA (ScrAB) showed similar basal and GIIS than mock cell transfected with pBS or untransfected cells (Fig. 1A) and comparable levels of cell death (less than 5%) assessed by trypan blue staining (data not shown).

For viral infection studies we reproduced as close as possible experimental conditions used in several ß-cell metabolism studies [27], [32], [38], [39], [40], in particular an IDHc knockdown study [27]. Ad-GFP and Ad-LacZ treatment of INS 832/13 cells with purified and rigorously titered viral stocks (see Experimental Procedures) was carried out. Cells infected at 10 MOI of Ad-LacZ or Ad-GFP showed a 2.5–5 fold increased basal insulin release and reduced insulin secretion in response to 5 mM and 10 mM glucose (Fig. 1B). Expression of the data as the amount of insulin released over basal value (1 mM glucose) indicated a 3–5 fold reduction in GIIS at 5 or 10 mM glucose in adenovirus-treated cells as compared to untransduced cells (Fig. 1 B, lower left panel). However Ad-LacZ or Ad-GFP infected cells at 10 MOI showed preserved KCl-induced insulin secretion and unchanged total insulin content (Fig. 1B). Thus, viral infection at 10 MOI of INS 832/13 cells induces some cell toxicity, with defective GIIS but not cell death or altered insulin content. Therefore, in subsequent work we refrained using viral transduction and carried out experiments in siRNA transfected cells.

**Figure 2:**
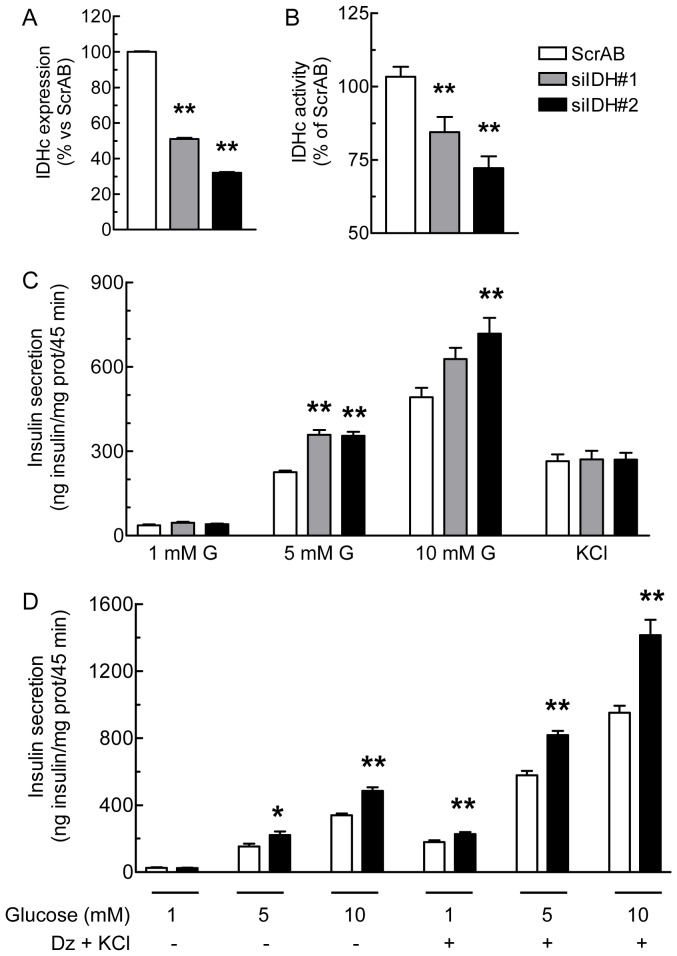
Knockdown of IDHc expression enhances glucose-induced insulin secretion. INS 832/13 cells were transfected with ScrAB, siIDHc#1 or siIDHc#2. A, IDHc mRNA expression level normalized with cyclophilin mRNA and presented as percentage vs the ScrAB condition. B, Enzymatic activity of IDHc normalized by protein content. C, Insulin secretion. Insulin release was measured in transfected cells incubated at 1, 5 or 10 mM glucose (G) or 1 mM glucose plus 35 mM KCl. D, Assessment of the amplification pathway of glucose-induced insulin secretion. Insulin secretion was measured in transfected cells incubated at 1, 5 or 10 mM glucose ±150 µM diazoxide plus 35 mM KCl (Dz+KCl). Insulin levels were normalized by protein content. Data represent the mean ± SEM of three to four independent experiments performed in quadruplicate. * *p*<0.05; ** *p*<0.01, vs ScrAB under the same condition, by one-way Anova, Dunnett's post-test.

***RNAi-knockdown of IDHc Expression Increases GIIS*** – The role of IDHc in the regulation of GIIS was studied using three siRNA directed against IDHc (siIDH#1-3) to knockdown this enzyme in INS 832/13 cells. In comparison with a combination of two scrambled control siRNA with no known targets (ScrAB), siIDH#1 and #2 effectively decreased IDHc mRNA expression, whereas siIDH#3 had little effect (not shown). IDH#1 and IDH#2 were therefore used in subsequent experiments. Forty hours post-transfection, siIDH#1 and siIDH#2 reduced IDHc mRNA level by about 50% and 70% (Fig. 2A) and significantly decreased IDHc activity by 15% and 30%, respectively (Fig. 2B). This reduction in enzymatic activity is likely underestimated due to contaminating mitochondrial IDH resulting from some mitochondrial damage and leakiness during cell homogenization and fractionation. However, the proof that IDHc knockdown was efficient is demonstrated by the fact that it resulted in increased levels of cellular isocitrate and NADP^+^ levels as predicted (see below). In contrast to a previous report using adenoviral constructs [27], RNAi knockdown of IDHc did not reduce but enhanced GIIS at 5 and 10 mM glucose, without affecting basal or KCl-induced insulin secretion (Fig. 2C). The cellular insulin content was unchanged under all conditions (8.1±0.8; 9.8±1.6 and 9.0±0.9 ng insulin/mg protein for ScrAB, siIDH#1 and siIDH#2, respectively).

We next investigated the involvement of the K_ATP_-independent/amplification pathway of GIIS using the combination of diazoxide (an activator of the K_ATP_ channels) with a stimulatory concentration of KCl (which allows permissive cytosolic Ca^2+^ level) [55]. As shown in Fig. 2D, insulin secretion at 5 and 10 mM glucose in the presence of KCl without or with diazoxide was markedly increased in the siIDH#2 condition as compared to the ScrAB control. These results indicate that knockdown of IDHc enhances the K_ATP_-independent/amplification pathway of GIIS.

**Figure 3:**
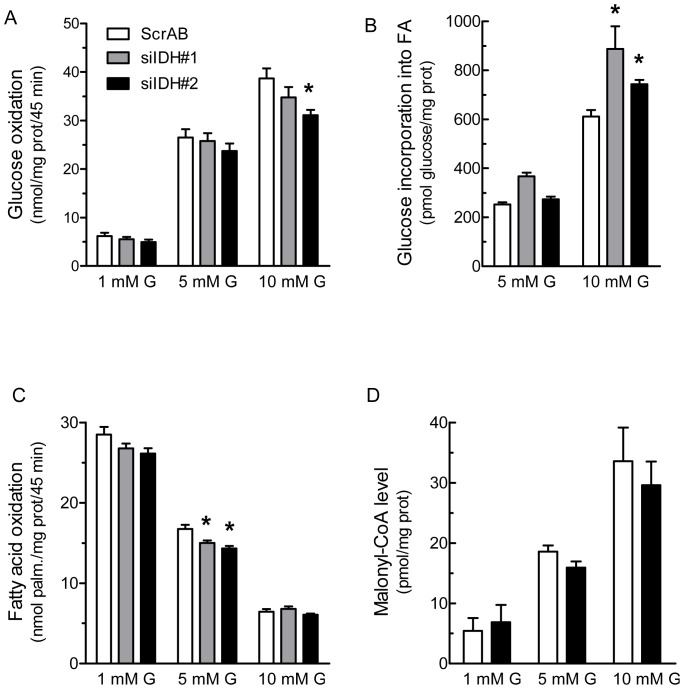
Reduction in IDHc expression alters fatty acid metabolism without affecting oxidative glucose metabolism. Transfected cells were incubated at 1, 5 or 10(G) and results were normalized by protein content. A, Glucose oxidation and B, Glucose incorporation into free fatty acids were monitored using [U-^14^C]glucose. C, Fatty acid oxidation was measured using [1-^14^C]palmitate. D, Malonyl-CoA levels determined using an enzymatic assay. Data represent means ± SEM of two (A) or three (B, C and D) independent experiments each performed in triplicate cell culture wells. * *p*<0.05; ** *p*<0.01, vs ScrAB under the same incubation condition, by one-way Anova, Dunnett's post-test.

***Downregulation of IDHc Affects Fatty Acid Metabolism*** – IDHc knockdown did not affect glucose oxidation rate at basal and intermediate glucose concentration. A small but significant decreased was measured at 10 mM glucose in siIDH#2 transfected cells (Fig. 3A).

As IDHc plays a role in the generation of cytosolic NADPH, a candidate metabolic coupling factor involved in *de novo* fatty acid synthesis, and because the pyruvate/citrate cycle is linked to fatty acid partitioning [56], we measured the incorporation of glucose into total fatty acids (using [U-^14^C]glucose) and fatty acid oxidation (using [1-^14^C]palmitate). Fig. 3B shows that IDHc knockdown by siIDH#1 or siIDH#2 increased glucose incorporation into fatty acids at 10 mM glucose. A similar increase was observed at 5 mM glucose concentration in siIDH#1 transfected cells. Fatty acid oxidation at 5 mM glucose was significantly decreased by siIDH#1 and siIDH#2, but not at 1 or 10 mM glucose (Fig. 3C). Malonyl-CoA levels increased in response to glucose concentration. However, knockdown of IDHc expression using siRNA#2 did not alter malonyl-CoA concentration (Fig. 3D).

**Figure 4:**
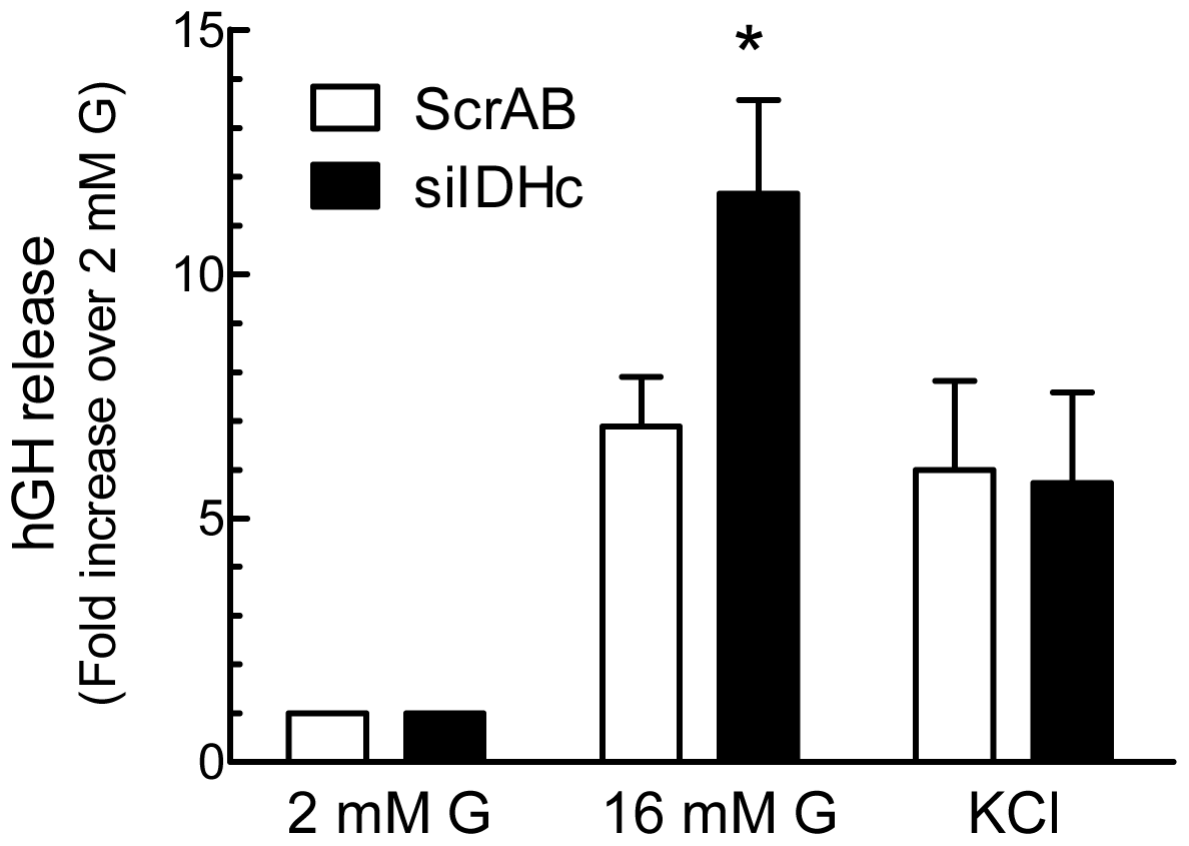
Knockdown of IDHc expression in dispersed rat islet cells increases glucose-induced hormone release. ScrAB and siIDH#2 (siIDHc) were co-transfected with human growth hormone (hGH) plasmid in dispersed rat islet cells. Cells were cultured for 48 h prior to the experiment. hGH release was measured in cells incubated at 2 or 16 mM glucose (G) or 2 mM glucose plus 35 mM KCl. hGH release was normalized by hGH cellular content and is expressed as fold increase over the 2 mM glucose condition. Data represent the mean ± SEM of three independent experiments performed in triplicate. * p<0.05 vs ScrAB under the same incubation condition by paired two-tailed Student *t* test.

***Islet Cells Studies*** – We examined if reducing IDHc expression in primary β-cells affects GIIS. Since transfection of whole islets or dispersed islet cells is not very efficient, we employed our most potent siRNA directed against IDHc (siIDH#2) in combination with human growth hormone (hGH), an established reporter for insulin release, since the cells that are transfected will capture simultaneously various constructs [48], [49]. Growth hormone is using the same vesicles for exocytosis as insulin itself. This method allowed us to measure the effect of IDHc KD on hormone release in transfected cells only, as opposed to insulin measurement that would have included both transfected and non-transfected cells. Therefore results for insulin release would not be truly quantitative and really informative if we had measured the parameters (KD and insulin secretion) in all the cells. Importantly we observe the same stimulatory effect (see below) on insulin secretion in transfected normal islet cells as in INS 832/13 cells. As shown in Fig. 4, dispersed rat islet cells transfected with hGH and control ScrAB efficiently secreted hGH in response to glucose and high KCl. The hGH release in response to glucose was increased in dispersed rat islet cells co-transfected with an siRNA against IDHc. Basal and KCl-induced hGH release were not affected by the IDHc siRNA.

**Figure 5:**
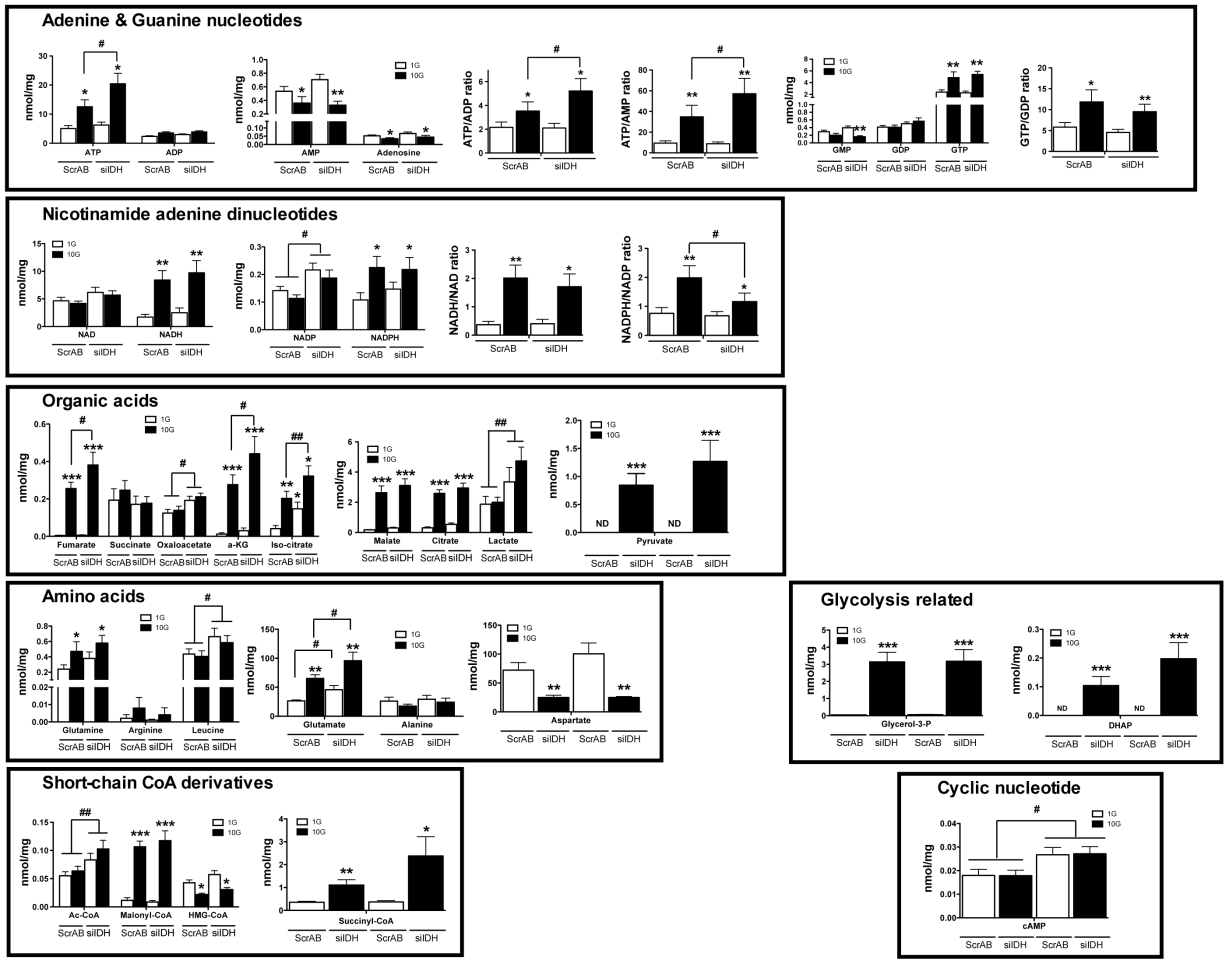
Targeted metabolomics of siRNA-transfected INS 832/13 cells. INS 832/13 cells were transfected with ScrAB and siIDH#2 (siIDH) and experimental conditions were similar as for insulin secretion. Cells were incubated at 1 (1 G) or 10 mM (10 G) glucose for 45 min. Results are presented by metabolite classes. Data represent the mean ± SEM of four independent experiments performed each with triplicate cell wells. * *p*<0.05; ** *p*<0.01; *** p<0.001 vs 1 G under the same transfection condition; # *p*<0.05; ## *p*<0.02, vs ScrAB under the same incubation condition by unpaired two-tailed Student *t* test.

***IDHc Knockdown Increases the Level of Metabolites Linked to Fuel-Induced Insulin Secretion*** – A targeted metabolomic study was undertaken to evaluate the cellular content of key metabolites that have been shown or are suspected to vary upon ß-cell activation by fuel stimuli. Using LC-MS/MS, we set up a methodology to quantitatively determine the level of 33 different metabolites in our model (Fig. 5). Several metabolites significantly increased in control ScrAB INS 832/13 cells in response to elevated glucose concentration (ATP, GTP, NADH, NADPH, fumarate, α-ketoglutarate, isocitrate, citrate, malate, pyruvate, glutamine, glutamate, glycerol-3-phosphate, dihydroxyacetone-phosphate, malonyl-CoA and succinyl-CoA), whereas others did not vary (ADP, GMP, GDP, NAD^+^, NADP^+^, succinate, oxaloacetate, lactate, arginine, leucine, alanine, acetyl-CoA and cAMP). AMP, adenosine, aspartate, and HMG-CoA levels decreased with glucose stimulation.

IDHc knockdown using siIDH#2 resulted as anticipated in increased cellular contents of both isocitrate and NADP^+^ at basal and elevated glucose (Fig. 5). Reduced expression of IDHc was associated at high glucose with increased ATP content and the ATP/ADP and ATP/AMP ratios, decreased NADPH/NADP^+^ ratio, higher levels of fumarate, oxaloacetate, α-ketoglutarate, lactate, leucine, glutamate, acetyl-CoA, and cAMP.

## Discussion

Using the siRNA technology in transfected cells, we observed that reduced IDHc expression results in stimulation of GIIS. This contrasts with a previous study showing that IDHc knockdown using an adenoviral approach reduces GIIS, a finding supporting a role for pyruvate/isocitrate/α-KG shuttle in ß-cell metabolic signaling [27]. Since in the present and in the previous studies the level of IDHc knockdown, the experimental conditions and the cellular models (INS 832/13 cells and rat islets) were similar [27], these opposite results are difficult to reconcile. How can this dichotomy be explained? The present study indicates that adenoviral constructs show some cell toxicity and are probably not an optimal tool to study metabolic fuel sensing in the ß-cell. This is of general interest to the ß-cell field since many groups, including us, have used this method. This is not to demise the ß-cell adenoviral work published so far but we wish to emphasize that performing appropriate controls of toxicity, metabolism and insulin secretion are mandatory in ß-cell transfection or transduction studies.

The Nucleofactor transfection method, instead of adenoviral infection, was chosen in the present study because it was without any apparent toxicity or unspecific effects on insulin secretion. This transfection technique did not affect basal secretion, KCl-induced insulin release, the total cellular insulin content or glucose oxidation, as indicated by comparison of the pBS and untransfected cells controls. In marked contrast, INS 832/13 cells infected with purified and well-titered adenoviruses at a relatively low MOI of 10 pfu/cell with two different control vectors (Ad-LacZ or Ad-GFP), showed defects in insulin secretion. In comparison to control cells, the infected cells showed high basal secretion, and GIIS was reduced by approximately 3 fold. Insulin content and KCl-induced insulin release, however, were not altered by adenoviral infection, suggesting that the decrease in GIIS observed in infected cells is not related to a marked general cellular toxicity, but is probably caused by an alteration of the mechanisms linking glucose metabolism to insulin vesicles exocytosis. Some studies also reported side effects of adenoviral infection on pancreatic β-cells. Adenoviral infection of MIN6 or INS 832/13 cells with an empty vector or a vector coding for β-galactosidase caused increased in caspase-3 activity, a marker of cell apoptosis [57]. In addition, adenoviral infection of rat, pig and human pancreatic islets with vectors coding for GFP or luciferase led to Akt1 activation and an increase in islet cell proliferation compared to non-infected islets [58]. Thus, we wish to put a note of caution on the use of the adenoviral approach in ß-cell studies, particularly related to metabolic signaling. We tentatively explain the difference between the results of the present study and those of the previous work on IDHc in the ß-cell [27] by a metabolic stress caused by adenoviruses *per se* that alterered normal ß-cell signaling for GIIS.

The data indicate that IDHc knockdown enhances GIIS at least in part by increasing the activity of the K_ATP_-independent/amplification pathway. The metabolic pathways and targeted metabolomic data provide an explanation as to how IDHc knockdown resulted in enhanced GIIS, particularly at intermediate glucose concentration at which overriding effects of very elevated glucose concentrations on metabolism are minimized. Thus, reducing IDHc expression resulted in increased levels of key metabolites that have been linked to ß-cell activation by fuels (several Krebs cycle intermediates) and that are either established (ATP, ATP/ADP ratio, cAMP) or candidate metabolic coupling factors in glucose signaling for secretion (glutamate, α-ketoglutarate, free fatty acids) [1].

How could these metabolites increase in response to IDHc knockdown to enhance GIIS? We verified the key prediction of an anticipated reduced flux through IDHc, that isocitrate and NADP^+^ levels should be increased and that the NADPH/NADP^+^ ratio should be reduced. In contrast, the single previous IDHc knockdown study did not measure isocitrate levels and reported an unexplained decreased levels at high glucose of both NADP^+^ and NADPH and a slight 15% reduction in the NADPH/NADP^+^ ratio [27].

**Figure 6:**
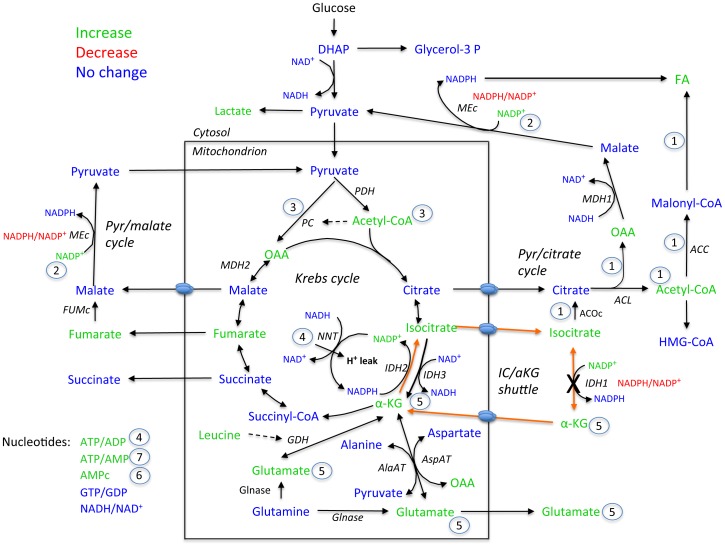
Schematic illustrating the metabolite changes induced by the reduction in IDHc expression. The numbers 1 to 7 refers to points mentioned in the discussion. AlaAT, alanine aminotransferase; AspAT, aspartate aminotransferase; ACC, acetyl-CoA carboxylase; ACL, ATP-citrate lyase; ACOc, cytosolic isoform of aconitase; DHAP, dihydroxyacetone phosphate; FFA, free fatty acids; FUMc, cytosolic isoform of fumarase; GDH, glutamate dehydrogenase; Glnase, glutaminase; Glycerol-3-P, glycerol-3-phosphate; α-KG, alpha-ketoglutarate; IC, isocitrate; IDH1 (or IDHc), cytosolic isoform of NADP^+^-dependent isocitrate dehydrogenase; IDH2, mitochondrial isoform of NADP^+^-dependent isocitrate dehydrogenase; IDH3, mitochondrial isoform of NAD^+^-dependent isocitrate dehydrogenase; MDH1, cytosolic isoform of malate dehydrogenase; MDH2, mitochondrial isoform of malate dehydrogenase; MEc, cytosolic isoform of malic enzyme; NNT, nicotinamide nucleotide transhydrogenase; OAA, oxaloacetate; PC, pyruvate carboxylase; PDH, pyruvate dehydrogenase; Pyr, pyruvate.

**Figure 7:**
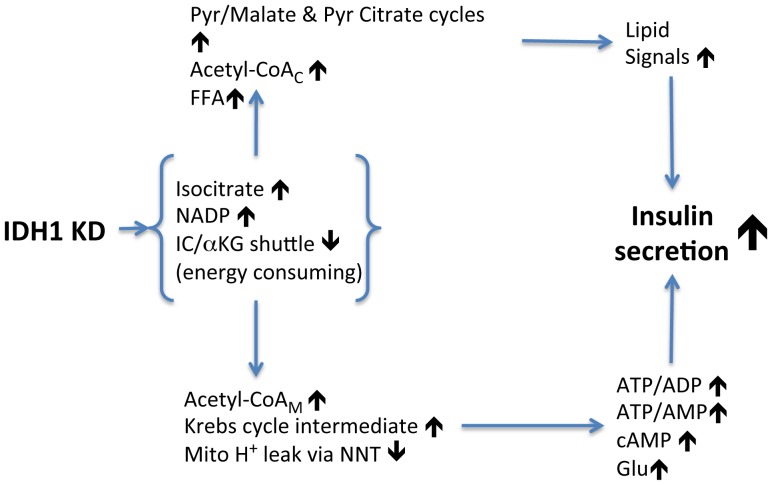
Model indicating how cytosolic isocitrate dehydrogenase knockdown results in enhanced glucose-induced insulin secretion. IC, isocitrate; α-KG, α-ketoglutarate; NNT, nicotinamide nucleotide transhydrogenase; Pyr, pyruvate.

The data provide a clear mechanism as to how IDHc knockdown enhances GIIS; by stimulating the production of several key metabolic coupling factors of insulin secretion (some Krebs cycle intermediates, acetyl-CoA, glutamate, cAMP and ATP). However the precise sequence of event(s) as how it does this is uncertain and we can only speculate as to how this might occur. We suggest below a cascade of events that is entirely compatible with our comprehensive study of metabolite measurements (see Figs. 6 and 7). In Fig. 6 the metabolites that increase, decrease or remain unchanged in IDHc knockdown cells vs control cells are color coded in green, red and blue, respectively. Reactions of the isocitrate/α-ketoglutarate shuttle are shown in orange. Also the sequence of events is numbered from 1 to 7. First, curtailing the isocitrate/α-ketoglutarate shuttle redirects isocitrate towards citrate raising cytosolic oxaloacetate and acetyl-CoA (Fig. 6). Acetyl-CoA is converted to malonyl-CoA which is then used together with NADPH for fatty acid synthesis, thus explaining the lack of a rise in malonyl-CoA and NADPH. An elevation in FFA would enhance GIIS via exocytotic effectors [59] or via their release from cells and GPR40 activation [60], [61] or indirectly via esterification processes and the synthesis of other lipid signaling molecules [62] (Fig. 7). Second, a rise in NADP^+^ would favor flux through cytosolic malic enzyme to sustain pyruvate/citrate and pyruvate/malate cycling. Third, a rise in mitochondrial acetyl-CoA, perhaps resulting from the sparing of carbons in the isocitrate/α-ketoglutarate shuttle and enhanced pyruvate cycling processes, will favor anaplerosis via pyruvate carboxylase activation [16] and enhance Krebs cycle activity, both contributing to accelerate ATP production. Fourth, also contributing to a rise in the ATP/ADP and ATP/AMP ratio is the fact that curtailing the isocitrate/α-ketoglutarate shuttle would reduce the demand on mitochondrial nicotinamide nucleotide transhydrogenase that synthesizes mitochondrial NADPH, thus reducing mitochondrial proton leak through the enzyme, and favoring ATP synthesis by proton leak through ATP synthase [34]. Interestingly, this would explain the lack of enhanced glucose oxidation and unaltered NADH/NAD^+^ ratio in IDHc knockdown cells when insulin secretion is enhanced. Fifth, enhanced anaplerosis will favor production of the candidate coupling factor glutamate [10] and α-ketoglutarate [63]. Sixth, a rise in ATP can favor cAMP production since there is evidence that variation in the level of ATP is coupled to changes in cAMP concentrations in ß-cells [64]. cAMP signaling is a potent amplification process for GIIS [65]. Seventh, the rise in ATP/ADP will promote Ca^2+^ signaling and the rise in ATP/AMP will reduce AMP-kinase activity, a negative regulator of insulin secretion [66].

The level of many of the measured metabolites was increased in IDHc siRNA treated cells and many were also unchanged but none of those measured were reduced. In control cells at elevated glucose, many metabolites were also increased or unchanged but the level of some metabolites was reduced. Glucose metabolism in ß-cells has unique features: glucose activation of metabolism is governed by substrate availability (a “push” rather than by a “pull” mechanism), which is not operative in most other tissues that contain low K_M_ hexokinases where metabolism primarily responds to hormonal stimulation, and energy expenditures of the tissue work load [67], [68]. This explains why many metabolites are increased upon glucose stimulation of the ß-cell. The results suggest that IDHc resulted in enhanced glycolysis as indicated by elevated levels of lactate (a marker of glycolysis) and acetyl-CoA, but that the extra-glucose carbons were metabolized via anaplerotic/cataplerotic pathways, as evidenced from metabolites determinations and enhanced incorporation of glucose into FFA. This might have occurred because of enhanced pyruvate/citrate cycling which reoxidizes NADH to NAD^+^ to sustain high glycolytic flux at the level of glyceraldehyde -3-dehydrogenase [21], [26].

We found that IDHc knockdown amplifies GIIS in the face of decreased NADPH/NADP^+^ ratio and constant total NADPH content. This does not discount a possible role of NADPH as a signaling molecule in GIIS. Thus, NADPH levels were higher at elevated glucose in both control and IDHc knockdown cells, and the extra secretion promoted by reducing expression of this enzyme likely resulted from the production of other metabolic coupling factors.

As mentioned in the introduction, the balance of the published work favours the view that metabolic flux and NADPH production via MEc is implicated in glucose signaling for insulin secretion in both tumoral and normal ß-cells. Herein, the inhibition of IDHc expression in INS 832/13 cells and rat islets stimulated insulin release. The opposite effect of MEc and IDHc knockdown on GIIS suggests that NADPH produced by MEc and IDHc may have different fates and roles in the pancreatic β-cell. Many studies in liver and adipose tissues have established the role of MEc in *de novo* lipogenesis [69]. Thus, the increase in glucose incorporation into fatty acids in INS 832/13 cells transfected with siIDHc is consistent with a possible role for MEc, through NADPH production, in the regulation of insulin secretion via lipogenesis and the production of lipid signaling molecules. These would include free fatty acids and some complex lipids, in particular diacylglycerol, involved in glycerolipid/FFA cycling [61]. However the contribution of enhanced *de novo* lipogenesis providing lipid signaling molecules for secretion is unlikely to be a major contributor to accelerated secretion upon IDHc KD in normal islets vs INS 832/13 cells, since we previously showed that fatty acid synthase is expressed at very low levels in normal islets relatively to other tissues but is well expressed in INS cells [70]. However it could become significant in diabetes, perhaps as a compensatory mechanism, since we showed that de novo lipogenesis and fatty acid synthase are induced in islet from ZDF rats [71].

With respect to IDH enzymes, both mitochondrial and cytoplasmic isoforms of NADP^+^-dependent IDH have been implicated in the control of the cellular redox state [72], [73]. Decreasing IDHc expression in three different cell lines (HeLa, HL-60 and NIH 3T3) led to a greater susceptibility to oxidative stress and apoptosis [74], [75], [76]. Perhaps, IDHc might play a role in the control of ß-cell growth and its redox state rather than in the positive signals for GIIS.

Most biological processes are regulated by both positive and negative modulations. So far, practically all the work aimed at defining the signaling pathways of GIIS has focused on activation processes, whereas little work has been done on the negative metabolic regulation of insulin secretion. Complementary to the fuel-induced generation of metabolic coupling factors, mechanisms likely exist to control insulin secretion through negative effectors. Inhibition of these negative pathways would enhance insulin secretion. Emerging evidences indicate that some metabolic pathways and enzymes negatively modulate GIIS. These include carnitine palmitoyltransferase 1, which catalyzes the limiting step of ß-oxydation [56], [77], short chain 3-hydroxyacyl-CoA dehydrogenase (SCHAD), which catalyzes the NAD^+^-dependent oxidation of short-chain 3-hydroxyacyl-CoA to 3-oxoacyl-CoA [78], [79], and a glucokinase/glucose-6-phosphatase futile substrate cycle [80]. Here we have provided strong evidences against the view that IDHc is an enzyme implicated in ß-cell activation of insulin secretion by glucose. However, we propose that flux through IDHc decelerates GIIS and that the isocitrate/α-ketoglutarate shuttle is a pathway that negatively modulates insulin secretion in response to glucose stimulation. Additional studies are required to determine how this pathway is regulated in health and diabetes and to precisely define the most important signals that amplify GIIS when metabolic flux through it is reduced.
